# Molecular Signals and Genetic Regulations of Neurological Disorders

**DOI:** 10.3390/ijms24065902

**Published:** 2023-03-21

**Authors:** Emanuele Rocco Villani, Emanuele Marzetti

**Affiliations:** 1 UOC Geriatra—Disturbi Cognitivi e Demenze, Dipartimento di Cure Primarie, AUSL Modena, 41012 Modena, Italy; 2 Department of Geriatrics and Orthopedics, Università Cattolica del Sacro Cuore, 00168 Rome, Italy; 3 Fondazione Policlinico Universitario A. Gemelli IRCCS, 00168 Rome, Italy

Neurological disorders are a large and heterogeneous field of research that can be tackled through a variety of approaches, ranging from epidemiology to molecular biology, through clinical, biostatistical, and laboratory experiments. The objective of this article collection was to gather the most recent evidence on signaling and genetic regulation of major neurological disorders (e.g., dementia, movement disorders, cerebrovascular disease, brain cancer). The articles included in the collection offer interesting insights into a range of biological processes involved in neurodegeneration. In addition, one contribution described an in silico approach to develop drugs for Parkinson’s disease (PD). Barrera-Vaquez et al., 2023 [1] sought to find alternative medications to monoamine oxidase B (MAO-B) inhibitors, that are extensively used to increase dopamine concentration in the brain in patients with PD. Despite their efficacy, MAO-B inhibitors have side effects that limit their use for an extended period of time. Through a data-driven approach, the authors searched for cheminformatic tools to find and optimize novel compounds with pharmacological activity against MAO-B (Figure 1). Afterward, the authors performed fingerprinting analysis and developed evolutionary libraries to obtain novel derived structures. These structures were evaluated through physiologically based pharmacokinetic modeling to eventually identify a molecule derived from rosmarinic acid with potential therapeutic value for PD.

Alzheimer’s disease (AD) is certainly the best known neurodegenerative disorder. It is characterized by deficits in multiple cognitive domains always including the memory domain and always involving neurodegeneration in the hippocampus. Several hypotheses have been proposed to explain the complex pathophysiology of AD, including altered neurogenesis and neuroinflammation. Olabiyi et al., 2021 [2] explored the possibility to boost adult hippocampal neurogenesis to obtain therapeutic gain in AD. Neurogenesis is mediated by neurotrophins, a family of secreted growth factors, of which nerve growth factor (NGF) and brain-NGF are the best known. Both are secreted as precursors and undergo proteolytic cleavage to become physiologically active. In mouse models of AD, such as the APP/PS1 model, pro-NGF was found to be increased compared with wildtype littermates. The authors found that p75NTR, the cellular receptor of pro-NGF, was downregulated in both human AD brains and the APP/PS1 model. Hence, the pro-NGF/p75NTR signaling pathway might serve as a new biological target for drug development.

The role of lipid-mediated inflammation in neurodegeneration was evaluated by Chen et al., 2022 [3]. The authors investigated the role of 12/15-lipoxygenase (12/15-LOX) in cognitive dysfunction associated with diabetes. They found that plasma levels of 12/15-LOX were higher in older adults with “diabetic cognitive impairment” (co-existence of diabetes and low scores on the Mini Mental State Examination (MMSE)) compared with both those with diabetes but average MMSE scores and normal controls. This finding was then confirmed in a murine model of diabetic rats. Hence, a 12/15-LOX inhibitor was administered to diabetic rats, with decreased levels of tumor necrosis factor alpha, interleukin (IL) 6, IL-12, and 12-hydroxyindoleic acid together with downregulation of 12/15-LOX, p38 mitogen-activated protein kinase (MAPK), amyloid peptide Aβ_1−42_, caspase 3, caspase 9, and cytosolic phospholipase A2. To further test their findings, the authors performed an in vitro experiment by exposing HT22 cells to a 12/15-LOX shRNA and a p38 MAPK inhibitor in high-glucose medium. The finding that the 12/15-LOX pathway plays a role in diabetic brain damage by activating p38 MAPK to promote inflammation and neuronal apoptosis, and that this mechanism could be inactivated by 12/15-LOX inhibitors is worth future research, perhaps not only limited to diabetic cognitive impairment.

In their contribution, Zhang et al., 2022 [4] studied the effects of prenatal exposure to systemic inflammation on offspring brain development and cerebral susceptibility to inflammatory insults eventually leading to cognitive decline in preclinical models. The authors found that prenatal maternal inflammation exacerbated lipopolysaccharide (LPS)-induced memory impairment, neuronal necrosis, and brain inflammatory response in offspring rats. The authors also reported a significant decrease in the exploratory behaviors of offspring rats that was meliorated by the administration of meloxicam, a ciclooxigenase-2 inhibitor. Collectively, the study by Zhang et al., 2022 [4] indicates that that lipid-mediated inflammation is involved in potentially revertible neurodegeneration. The study also shows that some mechanisms of neurodegeneration are already in place during intrauterine life. 

Jang et al., 2022 [5] studied LPS-induced striatal neurodegeneration in rats. LPS is an endotoxin found on the outer membrane of Gram-negative bacteria, which can induce oxidative stress, release of inflammatory cytokines, and eventually activate immune response. In the brain, LPS can activate resident inflammatory cells (microglia) and causes loss of striatal, hippocampal, and cortical neurons [6,7]. The novelty of the contribution relies in the fact that IL-4, an anti-inflammatory cytokine, was found to amplify the detrimental effect of LPS-induced neuroinflammation on striatal neurodegeneration by polarizing the microglia to produce proinflammatory molecules. Therefore, in the setting of neuroinflammation, the natural immune response can cause more damage than exogenous agents.

Neurodegeneration does not necessarily result in neuronal death. Indeed, brain cancer development can be a consequence of a neurodegenerative process that leads to loss of function, immortalization of cancer cells, and eventually, death of the individual. Oncogenesis shares pathways with neurodegeneration. For example, Chuang et al., 2021 [8] found that the upregulation of an E3 ubiquitin ligase named neuronal precursor cell-expressed developmentally downregulated 4-1 (NEDD4-1) reduced the expression tumor suppressors and enhanced the AKT/NFR2/HO-1 oxidative stress signaling axis, leading to higher glioblastoma resistance against temozolomide. Since glioblastoma is highly lethal with a recurrence rate of 90–95% in five years from diagnosis, the findings of Chuang et al., 2021 [8] offer new hints on possible novel therapeutic strategies.

Neurodegeneration is highly influenced by lifestyle habits, including alcohol (ab)use. A deeper understanding of the genetic basis of the risk of alcohol use and tendency to abuse is highly sought after. Al-Sabagh et al., 2022 [9] tested the link between circadian cycle and alcohol preference in mice. Interestingly, knock-out mice that did not express the gene for the circadian nuclear receptor REV-ERBα (a molecule known to disrupt molecular feedback loops integral to daily oscillations, and to impact diurnal fluctuations in the expression of proteins required for reward-related neurotransmission) showed a very low interest in drinking ethanol.

Which aspect of neurodegeneration should be addressed first?

## Figures and Tables

**Figure 1:**
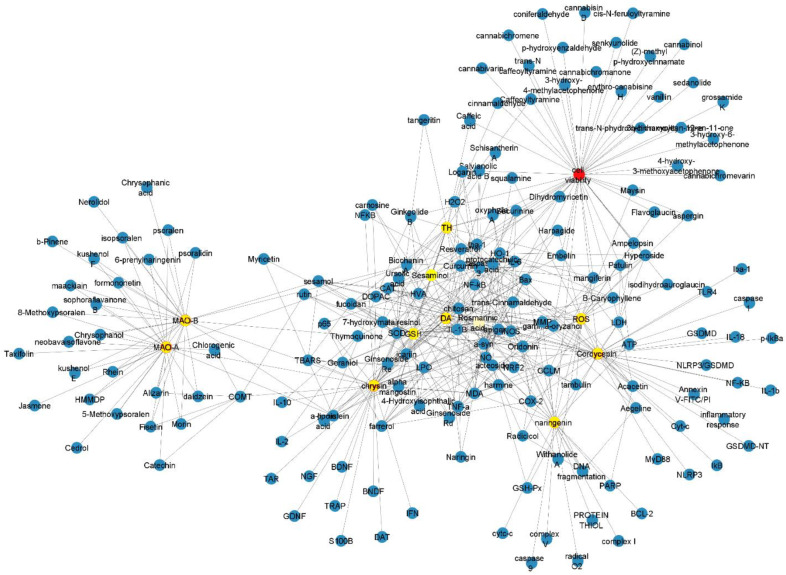
Structural network of the drugs used for Parkinson’s disease treatment, their pharmacological targets, and their representative components. The structural network shows the compounds (yellow nodes) and their pharmacological targets (blue nodes). Nodes represent the compounds and pharmacological targets, and edges indicate the reported interactions among them.

## References

[1] Barrera-Vazquez O., Santiago-de-la-Cruz J.A., Rivero-Segura N.A., Estrella-Parra E.A., Morales-Paoli G.S., Flores-Soto E., Gomez-Verjan J.C. (2023). Data-Driven Approaches Used for Compound Library Design for the Treatment of Parkinson’s Disease. Int. J. Mol. Sci..

[2] Olabiyi B.F., Fleitas C., Zammou B., Ferrer I., Rampon C., Egea J., Espinet C. (2021). proNGF Involvement in the Adult Neurogenesis Dysfunction in Alzheimer’s Disease. Int. J. Mol. Sci..

[3] Chen Q., Zheng Q., Yang Y., Luo Y., Wang H., Li H., Yang L., Hu C., Zhang J., Li Y. (2022). 12/15-Lipoxygenase Regulation of Diabetic Cognitive Dysfunction Is Determined by Interfering with Inflammation and Cell Apoptosis. Int. J. Mol. Sci..

[4] Zhang J., Yao P., Han W., Luo Y., Li Y., Yang Y., Xia H., Chen Z., Chen Q., Wang H. (2022). Maternal Prenatal Inflammation Increases Brain Damage Susceptibility of Lipopolysaccharide in Adult Rat Offspring via COX-2/PGD-2/DPs Pathway Activation. Int. J. Mol. Sci..

[5] Jang J., Hong A., Chung Y., Jin B. (2022). Interleukin-4 Aggravates LPS-Induced Striatal Neurodegeneration In Vivo via Oxidative Stress and Polarization of Microglia/Macrophages. Int. J. Mol. Sci..

[6] Flores-Martinez Y.M., Fernandez-Parrilla M.A., Ayala-Davila J., Reyes-Corona D., Blanco-Alvarez V.M., Soto-Rojas L.O., Luna-Herrera C., Gonzalez-Barrios J.A., Leon-Chavez B.A., Gutierrez-Castillo M.E. (2018). Acute Neuroinflammatory Response in the Substantia Nigra Pars Compacta of Rats after a Local Injection of Lipopolysaccharide. J. Immunol. Res..

[7] Zhao J., Bi W., Xiao S., Lan X., Cheng X., Zhang J., Lu D., Wei W., Wang Y., Li H. (2019). Neuroinflammation induced by lipopolysaccharide causes cognitive impairment in mice. Sci. Rep..

[8] Chuang H.Y., Hsu L.Y., Pan C.M., Pikatan N.W., Yadav V.K., Fong I.H., Chen C.H., Yeh C.T., Chiu S.C. (2021). The E3 Ubiquitin Ligase NEDD4-1 Mediates Temozolomide-Resistant Glioblastoma through PTEN Attenuation and Redox Imbalance in Nrf2-HO-1 Axis. Int. J. Mol. Sci..

[9] Al-Sabagh Y., Thorpe H.H.A., Jenkins B.W., Hamidullah S., Talhat M.A., Suggett C.B., Reitz C.J., Rasouli M., Martino T.A., Khokhar J.Y. (2022). Rev-erbα Knockout Reduces Ethanol Consumption and Preference in Male and Female Mice. Int. J. Mol. Sci..

